# Dual-Source Photon-Counting CT for Thoracic Arteries: Enhancing Image Quality and Diagnostic Assessability with Low-Energy Virtual Monoenergetic Imaging

**DOI:** 10.3390/diagnostics16193145

**Published:** 2026-09-28

**Authors:** Andreea I. Nica, Christian Booz, Giuseppe M. Bucolo, Leona S. Alizadeh, Thomas J. Vogl, Katrin Eichler, Vitali Koch, Leon D. Gruenewald, Ibrahim Yel, Scherwin Mahmoudi, Philipp Reschke, Jennifer Gotta, Daniel Dillinger, Hanns L. Kaatsch, Stephan Waldeck, Daniel Overhoff, Tommaso D’Angelo

**Affiliations:** 1 Division of Experimental Imaging, Department of Diagnostic and Interventional Radiology, University Hospital Frankfurt, Theodor-Stern-Kai 7, 60590 Frankfurt Am Main, Germany; andreea.nica10@yahoo.com (A.I.N.); boozchristian@gmail.com (C.B.); leona.alizadeh@outlook.de (L.S.A.); t.vogl@em.uni-frankfurt.de (T.J.V.); katrin.eichler@unimedizin-ffm.de (K.E.); vitali.koch@unimedizin-ffm.de (V.K.); leondavid.gruenewald@unimedizin-ffm.de (L.D.G.); dr.ibrahimyel@gmail.com (I.Y.); scherwin.mahmoudi@gmail.com (S.M.); philipp.reschke@outlook.de (P.R.); jennifergotta@aol.com (J.G.); 2 Department of Diagnostic and Interventional Radiology, University Hospital Frankfurt, Theodor-Stern-Kai 7, 60590 Frankfurt Am Main, Germany; 3 Diagnostic and Interventional Radiology Unit, BIOMORF Department, University Hospital “Policlinico G. Martino”, Via Consolare Valeria 1, 98100 Messina, Italy; giuseppebucolo94@gmail.com; 4 Department of Radiology and Neuroradiology, Bundeswehr Central Hospital, Rübenacher Straße 170, 56072 Koblenz, Germany; hannsleonhardkaatsch@bundeswehr.org (H.L.K.); stephanwaldeck@bundeswehr.org (S.W.); danieloverhoff@bundeswehr.org (D.O.); 5 Department of Vascular Surgery and Endovascular Surgery, Bundeswehr Central Hospital, Rübenacher Straße 170, 56072 Koblenz, Germany; danieldillinger@bundeswehr.org; 6 Department of Neuroradiology, University Medical Center Mainz, Langenbeckstraße 1, 55131 Mainz, Germany; 7 Department of Radiology and Nuclear Medicine, Medical Faculty Mannheim, University Medical Centre Mannheim, Heidelberg University, Theodor-Kutzer-Ufer 1-3, 68167 Mannheim, Germany

**Keywords:** thoracic arteries, virtual monoenergetic imaging, photon-counting CT, dual-energy CT

## Abstract

**Background/Objectives**: This study aimed to investigate the impact of low-energy virtual monoenergetic imaging (VMI) on quantitative and qualitative image characteristics, as well as its effect on the diagnostic assessability of thoracic arteries in photon-counting computed tomography angiography (CTA). **Methods**: We retrospectively evaluated 125 patients who underwent dual-source photon-counting CTA scans of thoracic arteries. We reconstructed standard CT images at 120 kV and VMI series at 15 keV intervals, ranging from 40 to 100 keV, and conducted quantitative and qualitative image analyses. For quantitative analysis, we assessed vascular CT numbers and contrast-to-noise ratio (CNR). For qualitative evaluation, three board-certified radiologists independently rated image quality, vascular contrast, and diagnostic assessability of the thoracic arteries using a five-point scale. **Results**: Quantitative image analysis revealed that 40 keV VMI reconstructions exhibited the highest mean CT value (HU: 1205 ± 286) and CNR (29.22 ± 9.13), followed by the 55 keV series (HU: 679 ± 161, CNR: 22.54 ± 7.36), significantly improved compared to higher keV levels and the standard 120 kV CT series (*p* < 0.001). Qualitative analysis showed the highest rating scores for 55 keV reconstructions, significantly higher than those of VMI series at higher energy levels and the standard 120 kV series (*p* < 0.001). **Conclusions**: VMI reconstructions at low energy levels (40–55 keV) significantly enhance vascular CTvalue and CNR, offering superior image quality and diagnostic assessability for thoracic arteries compared to standard CT series in photon-counting CTA.

## 1. Introduction

The thoracic arteries, encompassing the aorta, its branches, and the coronary arteries, are prone to various pathological conditions, many of which carry significant life-threatening risks. Thoracic aortic aneurysm and aortic dissection are among the most prevalent pathologies of the thoracic aorta [1]. With an estimated annual incidence of 5 per 100,000 individuals, most thoracic aortic aneurysms are incidentally detected during CT scans performed for unrelated indications [2,3]. If undiagnosed, these aneurysms may grow asymptomatically, eventually leading to aortic dissection, associated with a mortality rate of up to 49% in out-of-hospital settings, or rupture, where mortality exceeds 90% [2,4]. Similarly, coronary artery disease may remain clinically silent until the occurrence of myocardial infarction, while ischemic heart disease represents a leading cause of mortality worldwide, accounting for approximately 8.9 million deaths globally in 2015 [5,6]. Given these substantial risks, early detection of these conditions is crucial to reducing morbidity and mortality before the onset of acute cardiovascular events.

Computed tomography angiography (CTA) is the preferred diagnostic modality for assessing the thoracic arteries, as it provides comprehensive evaluation of vascular pathologies and is also well suited for follow-up imaging [7,8,9]. High image contrast is crucial for optimal visualization, influenced by factors such as the choice of contrast medium and patient-specific characteristics [10]. The development of dual-energy CT (DECT) has significantly enhanced cardiovascular imaging by enabling material decomposition and providing superior image quality [11,12,13,14,15].

Virtual monoenergetic imaging (VMI), a key innovation in DECT, has demonstrated substantial improvements in vascular imaging by enhancing iodine attenuation and reducing the volume of contrast medium required. Furthermore, VMI effectively mitigates beam hardening and blooming artifacts [11]. Low-energy VMI, leveraging photon energies close to the k-edge of iodine (33.17 keV), has been shown to increase CT attenuation and the contrast-to-noise ratio (CNR) [8,11,16]. Recent studies have indicated that low-energy VMI series provide the highest CNR compared to conventional polychromatic images, particularly in thoracic vascular imaging [8,17].

Further advancements in imaging have led to the introduction of photon-counting CT (PCCT), which surpasses DECT in diagnostic precision. PCCT employs energy-resolving photon-counting detectors, typically based on semiconductor materials such as cadmium telluride, that directly convert individual X-ray photons into electrical signals while preserving their energy information. This enables improved spatial resolution and reduced image noise, compared with conventional energy-integrating detectors, which first convert X-ray photons into visible light and subsequently into electrical signals [8,18,19,20,21]. A previous study by our research group investigated the use of low-energy VMI reconstructions for the visualization of the carotid arteries and demonstrated that 40–55 keV VMI significantly improved image quality, vascular contrast, and diagnostic assessability compared with standard CT series in photon-counting CTA [22]. Building on these findings, the present study extends the evaluation of low-energy VMI to the major thoracic arteries, including vessels of different calibers, an area that has not yet been comprehensively investigated. To our knowledge, this is the first study to investigate the application of low-energy VMI in PCCT across multiple major thoracic arteries.

The purpose of our study was to investigate the impact of low-keV VMI series on both quantitative and qualitative image parameters, as well as diagnostic assessability, for thoracic arteries in patients undergoing PCCTA.

## 2. Materials and Methods

### 2.1. Study Design

This retrospective study was approved by the ethics committee of our university hospital. The institutional review board (IRB) granted a waiver of informed consent due to the retrospective and observational nature of the study. Using our institutional databases, we identified patients who had undergone PCCTA between October 2020 and December 2022, prior to transcatheter aortic valve implantation (TAVI). We evaluated the ascending and descending aorta, brachiocephalic trunk, common carotid arteries, and coronary arteries. Exclusion criteria comprised: deviation from the standard CTA protocol (*n* = 9), the presence of metal artifacts (*n* = 1) and motion artifacts (*n* = 2) affecting the targeted vessels. Our final study cohort included 125 patients (Figure 1). Twenty arteries were excluded from the analysis due to the presence of bypass grafts, including three common carotid arteries (CCAs), five left main coronary arteries (LMs), five left anterior descending arteries (LADs), five circumflex arteries (CXs), and two right coronary arteries (RCAs). In two patients, all coronary arteries were excluded from the analysis. Accordingly, 122 CCAs, 120 LMs, 120 LADs, 120 CXs, and 123 RCAs were available for quantitative analysis.

### 2.2. CTA Examination and Image Reconstruction

All scans were performed using a dual-source PCCT scanner (NAEOTOM Alpha, Siemens Healthineers, Forchheim, Germany). The examination of the patients was performed in a supine position and was initiated by performing scout scans from the neck to the pelvis. Next, a non-enhanced ECG-gated heart scan was performed. For the angiographic phase, 80 mL of contrast medium (Xenetix 350 mgI/mL, Guerbet, Villepinte, France) was administered through a superficial forearm vein at a flow rate of 5 mL/s, followed by a 50 mL saline bolus at the same rate using a CT Motion power injector (Ulrich Medical, Ulm, Germany). The angiographic phase was bolus-triggered based on regions of interest (ROIs) placed in the thoracic arteries, covering vessels from the neck to the pelvis with a pitch of 3.2. Additionally, the acquisition was ECG-gated and automatically timed to capture the aortic valve data at 40% of the RR interval.

The obtained data was transferred to a post-processing workstation (syngo.via V70, Siemens Healthineers, Forchheim, Germany) where VMI series were reconstructed at varying virtual monoenergetic levels, ranging from 40 to 100 keV, in 15 keV intervals (Figure 2). The spectral post-processing datasets were examined using a 1.0 mm slice thickness and reconstructed with a 1.0 mm increment, applying a soft tissue convolution kernel (Qr40; Siemens). Additionally, standard images comparable to those obtained from a single-energy polychromatic 120 kV acquisition were reconstructed.

### 2.3. Quantitative Image Analysis

For the quantitative image evaluation, ROIs were drawn by an experienced radiologist within the lumens of the ascending (AA) and descending aorta (DA), common carotid artery (CCA), brachiocephalic trunk (BCT), left main coronary artery (LM), left anterior descending artery (LAD), circumflex artery (CX), and right coronary artery (RCA). A total of 10 ROIs were selected for each vessel, except for the CCA, where 5 ROIs were placed on each side. For each reconstruction, CT attenuation values (HU), standard deviation (SD), and CNR were extracted. To calculate the CNR, CT attenuation values were measured in the vessels and psoas muscle, while the standard deviation of an air ROI was used as the noise reference. The contrast-to-noise ratio (CNR) was calculated as the difference between the mean attenuation of the respective artery and the mean attenuation of the muscle ROI, divided by the standard deviation of the air ROI:

$$CNR=\frac{\left({HU}_{artery}-{HU}_{muscle}\right)}{{SD}_{air}}$$

We used the average values of all measurements for each vessel for the statistical analysis.

### 2.4. Qualitative Image Analysis

The qualitative image analysis was conducted by three board-certified radiologists (BLINDED), each with over six years of experience in vascular imaging. VMI series ranging from 40 to 100 keV, as well as the standard 120 kV series, were evaluated using a conventional picture archiving and communication system (PACS) workstation (Centricity, version 4.2; GE Healthcare, Solingen, Germany). The criteria for image assessment included overall image quality, vascular contrast, and the diagnostic assessability of each targeted artery. Each criterion was rated on a five-point Likert scale: 1 = very poor, 2 = poor, 3 = moderate, 4 = good, and 5 = optimal.

### 2.5. Statistical Analysis

We performed the statistical analysis using the statistical MedCalc software, version 23 (MedCalc Software Ltd., Ostend, Belgium). The Kolmogorov–Smirnov test was applied to assess the distribution of the data. For normally distributed data, comparisons across VMI reconstructions were made using repeated measures variance analysis, while the Friedman test was applied for non-normally distributed data. Continuous variables are expressed as mean values with standard deviation. A *p*-value of <0.05 was considered statistically significant.

For qualitative image analysis, we used Fleiss’ kappa test to evaluate the inter-rater agreement. A kappa value between 0.61 and 0.8 indicated substantial agreement, while values above 0.81 represented near-perfect agreement. A kappa score of 0 signified poor agreement, with ranges of 0 to 0.2 considered slight, 0.21 to 0.4 classified as fair, and 0.41 to 0.6 deemed moderate agreement.

## 3. Results

The final patient group included in our study consisted of 125 patients, of which 52 females (42%), with a mean age of 79 ± 7 years and 73 (58%) males, with a mean age of 79 ± 7 years, who underwent PCCT prior to TAVI.

We did not experience any complications during the examinations, and no CT scan was interrupted or had to be repeated. There were no deviations from the CT scan protocol among the patients included in the final study cohort. The mean cumulative CT dose index (CTDIvol) for all examinations was 5.96 ± 2.23 mGy, while the mean cumulative dose length product (DLP) was 398.3 ± 189.2 mGy.cm.

### 3.1. Quantitative Image Analysis

Regarding the CT attenuation measurements, the 40 keV VMI series provided the highest values, with an average for all thoracic arteries of 1205 ± 286 HU, followed by 55 keV with an average for all arteries of 679 ± 161 HU. The standard 120 kV series yielded significantly lower CT attenuation values, averaging 298 ± 67 HU across all thoracic arteries. Image noise was also highest in the 40 keV VMI series (112.23 ± 42.63) compared to the other reconstructions (all comparisons: *p* < 0.001).

Furthermore, the 40 keV VMI series exhibited the highest mean CNR values (29.22 ± 9.13) across all vessels among all reconstructions (all comparisons: *p* < 0.001). We observed a gradual decrease in CNR with increasing energy levels in the VMI reconstructions for each evaluated artery. The greatest increases in CNR compared with standard 120 kV imaging were observed in the larger and intermediate-sized arteries, while the coronary arteries also demonstrated a substantial increase.

The obtained average values for all VMI series and standard CT series are displayed in Table 1 and Figure 3.

### 3.2. Qualitative Image Analysis

The qualitative image analysis demonstrated the highest rating scores for the 55 keV reconstructions, with an overall image quality of 4.5 ± 0.2, vascular contrast of 4.5 ± 0.4, and diagnostic assessability of chest vessels at 4.1 ± 0.4. Substantial inter-rater agreement was observed, with a Kappa value of 0.74 (95% CI: 0.58–0.92). The 55 keV reconstructions were followed by the 70 keV series, which achieved overall image quality ratings of 4.3 ± 0.2, vascular contrast of 4.2 ± 0.3, and diagnostic assessability of chest vessels at 4.0 ± 0.6. For the 70 keV series, an almost perfect inter-rater agreement was noted, with a Kappa value of 0.84 (95% CI: 0.71–0.95) (Table 2).

In contrast, the standard 120 kV reconstructions showed lower scores, with overall image quality at 3.6 ± 0.4, vascular contrast at 3.4 ± 0.3, and diagnostic assessability of chest vessels at 3.3 ± 0.7. Substantial inter-rater agreement was also noted, with a Kappa value of 0.78 (95% CI: 0.53–0.88).

The qualitative analysis revealed significantly higher scores for the 40–70 keV reconstructions compared to both higher-energy VMI series and the standard 120 kV series (*p* < 0.001). Among the 40–70 keV reconstructions, most comparisons were statistically significant, except for vascular contrast between the 40 and 70 keV series and diagnostic assessability between the 55 and 70 keV reconstructions (*p* > 0.05).

## 4. Discussion

Our results demonstrate superior image quality in low-energy VMI reconstructions across both qualitative and quantitative analyses. Quantitative analysis revealed that the 40 keV VMI series showed the highest mean CT attenuation (1205 ± 286 HU) and CNR (29.22 ± 9.13). The 55 keV series followed, showing significantly elevated parameters compared to higher-energy VMI and standard polychromatic 120 kV images. Qualitative analysis similarly favored low-energy VMI reconstructions, with 55 keV receiving the highest ratings for overall image quality, vascular contrast, and diagnostic assessability.

While the 40 keV series exhibited the best quantitative parameters, the increased image noise at this energy level reduced its subjective ratings. The 55 keV reconstructions, with balanced CT attenuation and reduced noise, achieved the highest subjective scores (overall image quality: 4.5 ± 0.2; vascular contrast: 4.5 ± 0.4; diagnostic assessability: 4.1 ± 0.4). The second-highest qualitative ratings were observed for the 70 keV series. Statistical analysis confirmed significant differences between most energy levels, except for vascular contrast between 40 and 70 keV and diagnostic assessability between 55 and 70 keV reconstructions (*p* > 0.05).

We observed the greatest improvements in CNR in the large and intermediate-sized arteries. Nevertheless, substantial improvements compared with standard 120 kV imaging were also observed in the coronary arteries, suggesting that low-keV VMI can enhance image quality across a broad range of vessel sizes, including smaller vessels.

Our study demonstrated statistically significant results, which align with existing literature on the benefits of low-energy VMI reconstructions using PCCT [22]. VMI series at low-keV levels (40–70 keV) have been shown to enhance image quality in cardiovascular imaging by improving quantitative parameters such as CNR. These improvements are largely attributed to the increased iodine signal, as these energy levels are close to the k-edge of iodine [8,11,16]. Studies investigating this aspect date back to 2013/2014. For instance, Apfaltrer et al. [18] investigated the use of VMI at low-keV levels in CT pulmonary angiography. Their findings demonstrated that vessel attenuation and CNR in 70 keV series were superior to both other monoenergetic reconstructions and standard 120 kV polyenergetic datasets. Their results also suggested that incorporating low-energy VMI series into clinical practice could reduce the amount of contrast medium required for DECT-CT pulmonary angiography examinations. Similarly, Delesalle et al. [23] investigated the use of monochromatic imaging with DECT for visualizing chest vessels using low concentrations of contrast material. They found that optimal imaging was achieved at 60 keV and 100 keV. These findings emphasize the potential of low-energy VMI to minimize contrast medium usage, which is particularly advantageous for patients with renal impairment, diabetes mellitus, or other contraindications to contrast agents [24,25,26,27]. By lowering the exposure to contrast material, VMI may offer an opportunity to reduce contrast exposure, particularly in patients at increased risk of renal impairment. However, whether reduced contrast administration translates into a lower risk of contrast-associated acute kidney injury remains uncertain and warrants further investigation [28,29,30,31,32].

VMI also potentially allows for optimizing image quality based on patient-specific characteristics, such as BMI (body mass index), age, and medical condition, for example, by adjusting the energy levels to achieve high image contrast with low image noise in patients with high BMI values [33]. This approach could also help improve CT scan protocols by enabling high-quality images with minimized artifacts and enhanced contrast, all while potentially reducing radiation doses through improved CNR and the possibility to lower tube voltage in CT scans [34]. However, further investigation into its applicability in clinical settings is required and should be explored in future studies.

In acute vascular emergencies such as aortic dissection or pulmonary embolism, achieving adequate vascular contrast can be challenging due to factors such as reduced cardiac output and vasoconstriction. Low-energy VMI reconstructions, with improved CT attenuation and CNR, may address these challenges, offering better visualization of true and false lumens in aortic dissections and enhancing diagnostic reliability [8,17,18,22].

Recent studies on VMI with PCCT corroborate our findings. Sartoretti et al. [8] reported that 40 keV VMI provided optimal image quality for coronary artery visualization, achieving high scores in both objective and subjective analyses. Similarly, in our previous study of the carotid arteries, 40 keV VMI provided the best quantitative parameters for carotid artery imaging, whereas 55 keV reconstructions achieved the highest subjective image quality ratings [22].

The emergence of PCCT has brought numerous advantages to vascular assessment, including enhanced spatial and temporal resolution, as well as applications such as VMI and VNC (Virtual Non-Contrast Imaging) [8,19,20,35]. VMI enhances image quality, while VNC can replace traditional native CT scans, significantly reducing radiation exposure without compromising diagnostic accuracy. Additionally, spectral imaging plays a crucial role by enabling the differentiation of materials based on their energy levels, further improving diagnostic precision.

Our study has several limitations that should be considered. One of the primary limitations is the retrospective nature of the study design, which may have impacted the results. Additionally, the process of delineating ROIs proved challenging in some cases due to the presence of vascular pathologies such as calcifications and soft plaques, which may have influenced the accuracy of the analysis. Furthermore, the energy levels selected for analysis (ranging from 40 to 100 keV, with intervals of 15 keV) were chosen arbitrarily, and different results might be observed at untested energy levels. We also evaluated the standard contrast injection protocol, using a standard contrast medium volume of 80 mL. However, as no cohort receiving a reduced amount of contrast medium was available in our retrospective dataset, we could not perform a direct comparison. This aspect is highly clinically relevant and warrants further investigation in future studies.

### Conclusions

In conclusion, low-energy VMI reconstructions with PCCT, particularly at 40–55 keV, demonstrate significant improvements in both quantitative and qualitative image quality. The 40 keV series achieved the highest quantitative parameters, while the 55 keV reconstructions provided superior subjective ratings. These findings suggest that PCCT VMI reconstructions at 40–55 keV offer a viable strategy for optimizing thoracic artery imaging, outperforming standard 120 kV reconstructions and highlighting the clinical potential of this advanced imaging technology.

## Figures and Tables

**Figure 1:**
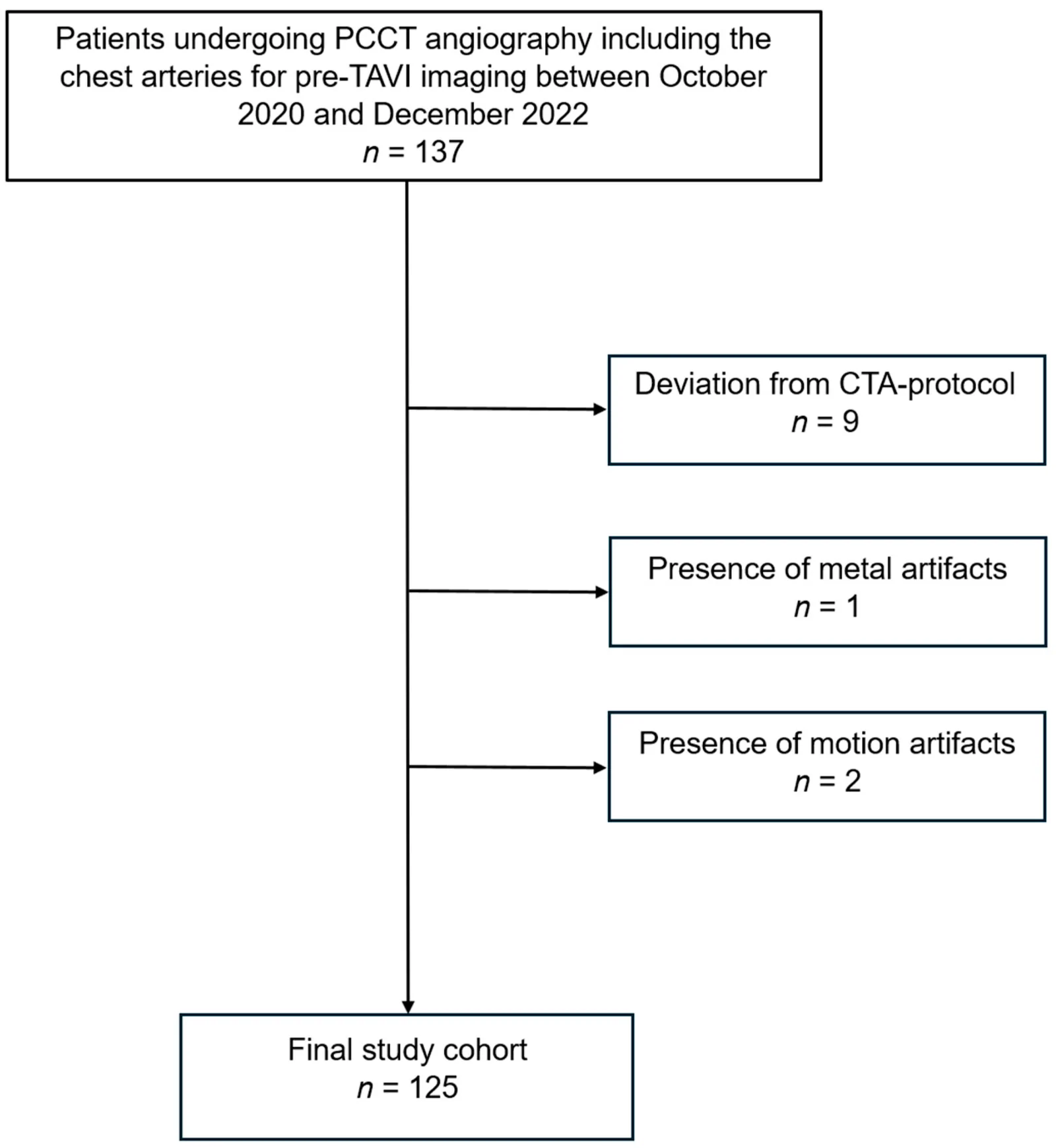
Graphical representation of the inclusion and exclusion criteria of the study group.

**Figure 2:**
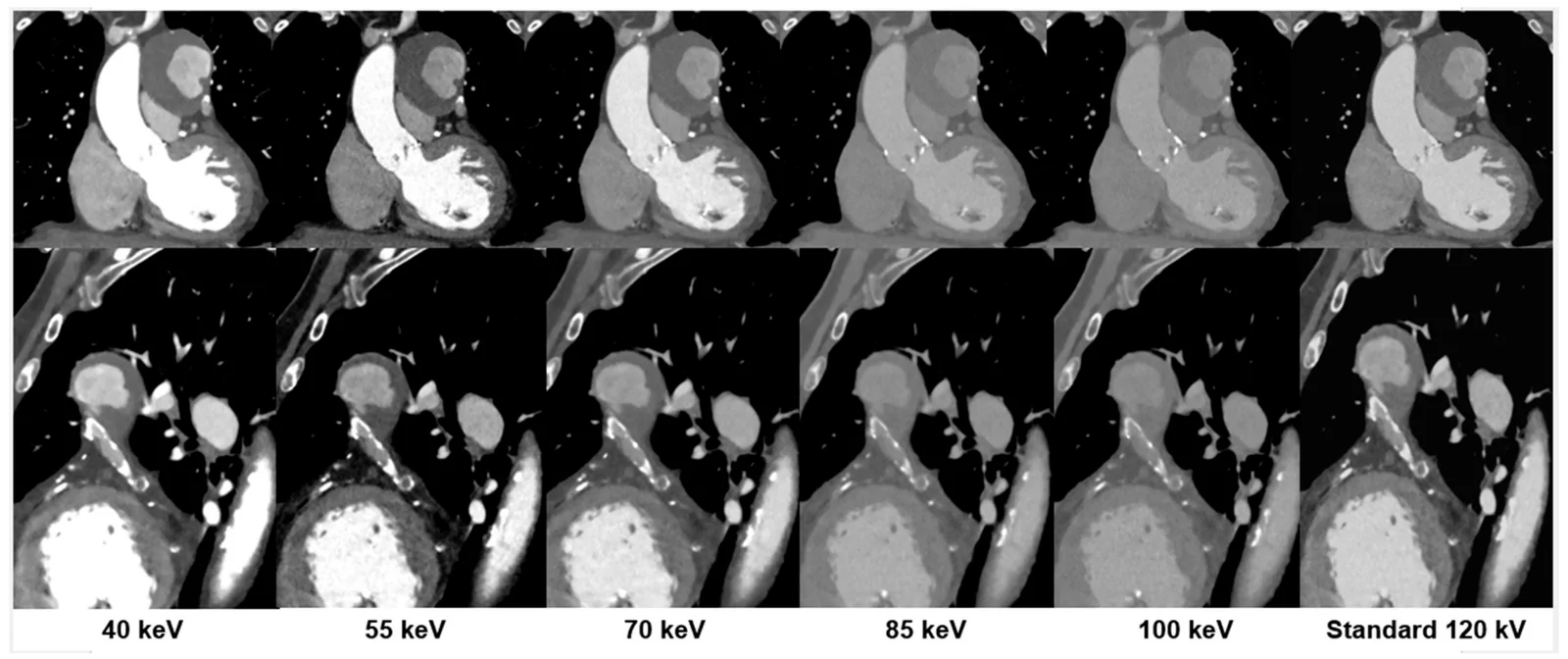
Dual-source photon-counting CTA in a male patient with an aneurysm of a coronary bypass and poor contrast in the bypass displayed in coronal and sagittal images. Low-energy VMI+ reconstructions at 40 keV provide highest iodine signal in the perfused lumen of the proximal portion of the bypass. Additionally, the occlusion in the distal part of the bypass is displayed with higher contrast to the perfused lumen in the proximal part of the bypass in comparison with higher keV VMI+ levels and the standard 120 kV CT series.

**Figure 3:**
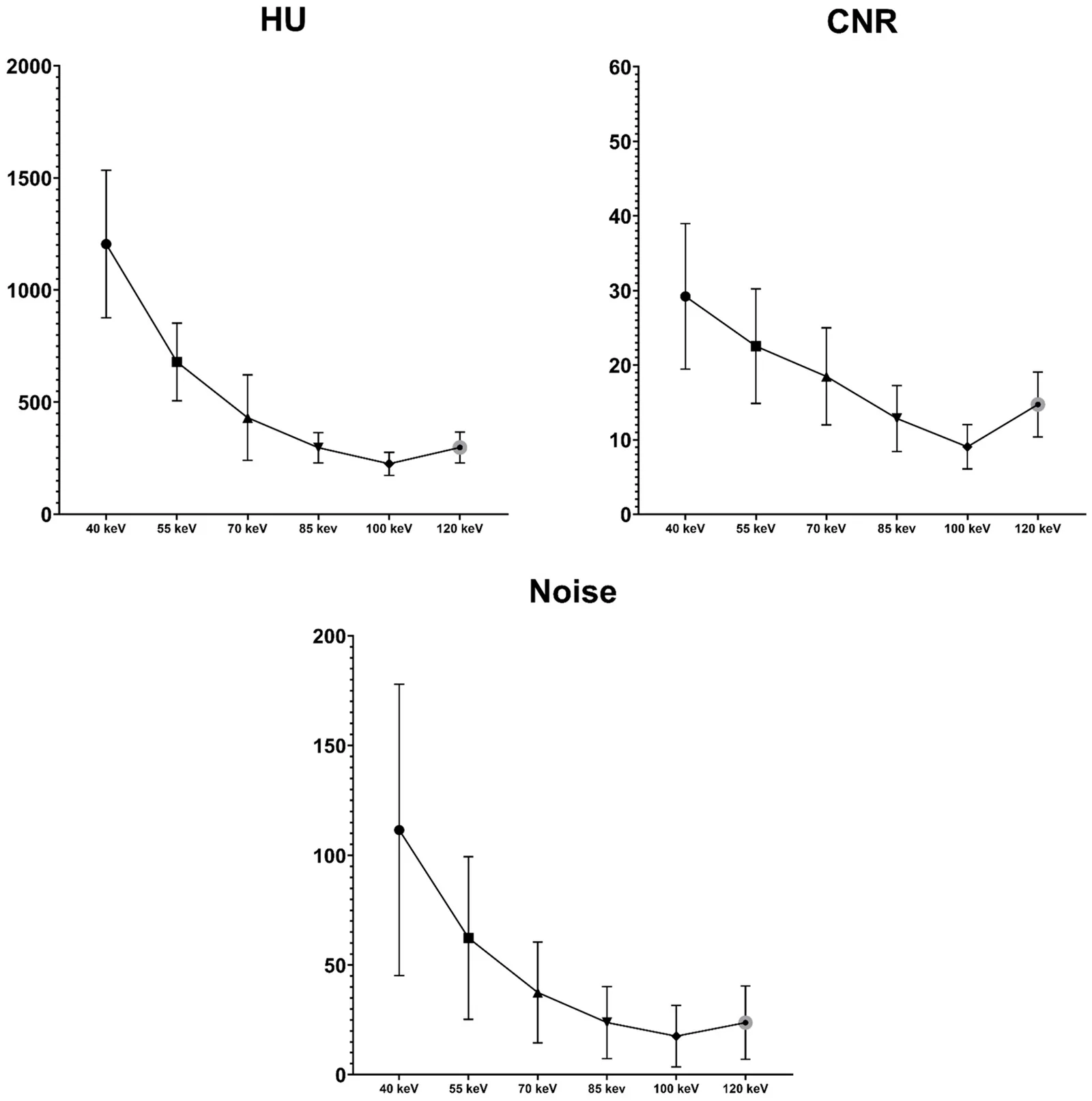
Graphical representation of the mean values of HU (Hounsfield units), CNR (contrast-to-noise ratio), and image noise for all vessels. The highest values for all analyzed parameters included in the quantitative image analysis were observed at 40 keV (HU: 1205 ± 286; CNR: 29.22 ± 9.13; image noise: 112.23 ± 42.63). A gradual decrease in all parameters was observed with increasing energy levels.

**Table 1:** Results of the quantitative measurements in all arteries expressed in mean values and standard deviations.

Parameters	40 KEV	55 KEV	70 KEV	85 KEV	100 KEV	Standard 120 KV
**Reconstructed HU**						
**AA**	1298 ± 350	723 ± 179	444 ± 104	303 ± 64	225 ± 43	304 ± 68
**AD**	1322 ± 310	737 ± 167	457 ± 97	315 ± 62	235 ± 43	316 ± 65
**CCA**	1358 ± 299	751 ± 160	460 ± 93	312 ± 60	229 ± 43	314 ± 61
**BCT**	1337 ± 323	738 ± 183	458 ± 101	313 ± 65	232 ± 45	311 ± 62
**LM**	1161 ± 287	607 ± 158	415 ± 98	292 ± 69	224 ± 53	292 ± 68
**LAD**	1052 ± 143	606 ± 143	388 ± 91	281 ± 73	220 ± 64	283 ±76
**CX**	1017 ± 306	593 ± 155	381 ± 91	269 ± 65	209 ± 53	274 ± 67
**RCA**	1077 ± 263	618 ± 143	393 ± 89	282 ± 67	220 ± 58	283 ± 69
**Average of all arteries**	**1205 ± 286**	**679 ± 161**	**431 ± 144**	**297 ± 66**	**225 ± 51**	**298 ± 67**
**Image Noise**	112 ± 43	63 ± 23	38 ± 14	24 ± 10	18 ± 10	24 ± 10
**CNR**						
**AA**	31.68 ±10.64	24.11 ± 8	19.43 ± 6.85	13.26 ± 4.39	9.11 ± 2.73	15.08 ± 4.53
**AD**	32.20 ± 9.54	24.62 ± 7.72	20.13 ± 6.79	13.84 ± 4.34	9.62 ± 2.77	15.69 ± 4.52
**CCA**	33.10 ± 9.76	25.02 ± 7.38	20.39 ± 6.8	13.67 ± 4.27	9.35 ± 2.77	15.60 ± 4.14
**BCT**	32.61 ± 9.91	24.65 ± 8.25	20.13 ± 6.85	13.77 ± 4.47	9.45 ± 2.78	15.37 ± 4.04
**LM**	28.07 ± 8.51	21.86 ± 7.49	17.99 ± 6.23	12.56 ± 4.24	9 ± 2.93	14.45 ± 4.17
**LAD**	25.38 ± 8.14	20.03 ± 6.83	16.68 ± 5.9	11.97 ± 4.56	8.80 ± 3.44	14.04 ± 4.52
**CX**	24.36 ± 8.65	19.54 ± 6.9	18.84 ± 30.02	11.44 ± 4.25	8.23 ± 2.94	13.58 ± 4.33
**RCA**	26.02 ± 7.88	20.33 ± 6.29	16.84 ± 5.5	12.13 ± 4.24	8.82 ± 3.15	13.92 ± 4.05
**Average of all arteries**	**29.22 ± 9.13**	**22.54 ± 7.36**	**18.81 ±9.37**	**12.84 ± 4.35**	**9.05 ± 7.55**	**14.71 ± 4.29**

Abbreviations: AA: ascending aorta; DA: descending aorta; CCA: common carotid artery; BCT: brachiocephalic trunk; LM: left main coronary artery; LAD: left anterior descending artery; CX: circumflex artery; RCA: right coronary artery; HU: Hounsfield units; CNR: contrast-to-noise ratio.

**Table 2:** Average results of the qualitative image analysis of all arteries. All data are presented as mean values with standard deviations.

keV	Overall Image Quality	Vascular Contrast	Diagnostic Assessability	Kappa Value	*p*-Value
**40 keV**	4.1 ± 0.3	4.2 ± 0.4	3.8 ± 0.6	0.82 (95% CI: 0.64–0.96)	40–70 keV, *p* >0.05 (vascular contrast) Others, *p* < 0.001
**55 keV**	4.5 ± 0.2	4.5 ± 0.4	4.1 ± 0.4	0.74 (95% CI: 0.58–0.92)	55–70 keV, *p* > 0.05 (diagnostic assessability) Others, *p* < 0.001
**70 keV**	4.3 ± 0.2	4.2 ± 0.3	4.0 ± 0.6	0.84 (95% CI: 0.71–0.95)	70–40 keV, *p* >0.05 (vascular contrast) 70–55 keV, *p* > 0.05 (diagnostic assessability) Others, *p* < 0.001
**85 keV**	3.3 ± 0.3	3.1 ± 0.5	3.2 ± 0.4	0.81 (95% CI: 0.64–0.93)	All, *p* < 0.001
**100 keV**	2.5 ± 0.6	2.5 ± 0.7	2.1 ± 0.5	0.81 (95% CI: 0.66–0.93)	All, *p* < 0.001
**Standard 120 kV**	3.6 ± 0.4	3.4 ± 0.3	3.3 ± 0.7	0.78 (95% CI: 0.53–0.88)	All, *p* < 0.001

## Data Availability

The data supporting the findings of this study are available from the corresponding author upon reasonable request.

## Abbreviations

AA	Ascending aorta
DA	Descending aorta
BCT	Brachiocephalic trunk
BMI	Body mass index
CCA	Common carotid artery
CNR	Contrast-to-noise ratio
CTA	Computed tomography angiography
CX	Circumflex artery
DECT	Dual-energy CT
HU	Hounsfield units
IRB	Institutional Review Board
LAD	Left anterior descending artery
LM	Left main coronary artery
PCCT	Photon-counting CT
RCA	Right coronary artery
ROIs	Regions of interest
TAVI	Transcatheter aortic valve implantation
VMI	Virtual monoenergetic imaging
VNC	Virtual Non-Contrast Imaging
